# Fusing multiple protein-protein similarity networks to effectively predict lncRNA-protein interactions

**DOI:** 10.1186/s12859-017-1819-1

**Published:** 2017-10-16

**Authors:** Xiaoxiong Zheng, Yang Wang, Kai Tian, Jiaogen Zhou, Jihong Guan, Libo Luo, Shuigeng Zhou

**Affiliations:** 1 Shanghai Key Lab of Intelligent Information Processing, and School of Computer Science, Fudan University, 220 Handan Road, Shanghai, 200433 China; 2 The Bioinformatics Lab at Changzhou NO. 7 People’s Hospital, Changzhou, Jiangsu, 213011 China; 3 Department of Computer Science and Technology, Tongji University, 4800 Cao’an Road, Shanghai, 201804 China; 4 The institute of subtropical Agriculture, China Academy of Sciences, 444 Yuandaer Road, Mapoling, Changsha, 410125 China; 5 School of Software, Jiangxi Normal University, 99 Ziyang Avenue, Nanchang, 330022 China

**Keywords:** lncRNA-Protein Interaction, Random walk, Similarity network fusion

## Abstract

**Background:**

Long non-coding RNA (lncRNA) plays important roles in many biological and pathological processes, including transcriptional regulation and gene regulation. As lncRNA interacts with multiple proteins, predicting *lncRNA-protein interactions* (lncRPIs) is an important way to study the functions of lncRNA. Up to now, there have been a few works that exploit *protein-protein interactions* (PPIs) to help the prediction of new lncRPIs.

**Results:**

In this paper, we propose to boost the prediction of lncRPIs by fusing multiple *protein-protein similarity networks* (PPSNs). Concretely, we first construct four PPSNs based on protein sequences, protein domains, protein GO terms and the STRING database respectively, then build a more informative PPSN by fusing these four constructed PPSNs. Finally, we predict new lncRPIs by a random walk method with the fused PPSN and known lncRPIs. Our experimental results show that the new approach outperforms the existing methods.

**Conclusion:**

Fusing multiple protein-protein similarity networks can effectively boost the performance of predicting lncRPIs.

## Background

Long non-coding RNAs (lncRNAs in short), one type of non-protein coding transcripts longer than 200 nucleotides, play important roles in complex biological processes, ranging from transcriptional regulation, epigenetic gene regulation to disease identification [1]. Up to date, a number of lncRNAs have been identified, such as HOTAIR [2], MALAT-1 [3] and Xist [4], but most of them are still unknown. Researches have shown that most lncRNAs can exert their functions by interfacing with multiple corresponding RNA binding proteins [5]. Therefore, predicting *lncRNA-protein interactions* (lncRPIs) is an important way to study the functions of lncRNAs.

In the literature, there are more and more works that employ machine learning methods to predict the interactions between RNAs/ncRNAs/lncRNAs and proteins. For example, Muppirala et al. [6] proposed the RPISeq method for identifying *RNA-protein interactions* (RPIs) by using Random Forest (RF) and Support Vector Machine (SVM) classifiers trained with features of protein and RNA sequences. Bellucci et al. [7] proposed the catRAPID method by using a number of physicochemical features, including hydrogen bonding, van der Waals interaction and secondary structure, for predicting lncRPIs. Wang et al. [8] developed an extended Naive Bayes classifier for predicting RPIs using only protein and RNA sequence information. Lu et al. [9] developed the LncPro tool for lncRPI prediction based on Van der Waals propensities, hydrogen bonding and secondary structures extracted from lncRNA-protein pairs.

Cheng et al. [10] proposed the PRIPU approach that uses only positive and unlabeled examples to predict RPIs. Recently, Suresh et al. [11] employed SVM to predict ncRNA-protein interactions (ncRPIs) by using the information of sequences and predicted structural peculiarities of proteins and RNAs, and Cheng et al. [12] proposed to boost the performance of protein-RNA interaction prediction by selecting high-quality negative samples.

In addition, there are some works that identify lncRPIs from the perspective of network. That is, to construct networks by using known lncRPIs and PPIs as well as lncRNA-lncRNA interactions. For example, Yang et al. [13] first constructed a heterogeneous network of lncRNAs and proteins, and then employed HeteSim [14] — a pair-wise random walk model that can evaluate n between heterogeneous objects, to evaluate the connection possibilities between lncRNAs and proteins in the network, and thus identify new lncRPIs. However, in their heterogeneous network, PPIs were represented in 0/1 style. That is, if two involved proteins interact, there is an edge between them with weight 1; Otherwise there is no edge. Li et al. [15] proposed the LPIHN method to infer new lncRPIs, which is roughly similar to the method above. They also constructed a heterogenous network of lncRNAs and proteins. But the LPIHN method is different from the method of Yang et al. [13] in three aspects: 1) lncRNA-lncRNA interactions are considered in the heterogenous network; 2) PPIs are represented by protein-protein similarity based on the PPI confidence data from the STRING database; and 3) the random walk with restart model (instead of HeteSim) was used. One common point of these two works is that the only used protein-related information was PPI data

In this paper, to boost the performance of lncRPI prediction we propose to fuse multiple protein-protein similarity networks (PPSNs), and integrate the fused PPSN with known lncRPIs to construct a more informative heterogeneous network, on which new lncRPIs are inferred. Concretely, we first use protein sequences, protein domains, GO terms and the STRING database respectively to construct four PPSNs, then employ the *similarity network fusion* (SNF) algorithm [16] to combine the four PPSNs into a fused PPSN. Following that, a heterogeneous lncRNA-protein network based on the fused PPSN and known lncRPIs is built. Finally, the HeteSim algorithm is performed on the heterogeneous lncRNA-protein network to infer new lncPRIs. Extensive experiments show that our approach outperforms not only the existing method but also those using only one PPSN.

## Methods

In this section, we introduce the lncRPI data used in our study and present the details of our method.

### Datasets

We extracted Homo sapiens ncRNA-protein interactions from NPInter (v2.0) [17] and Homo sapiens lncRNA from the ncRNA database NONCODE (v4.0) [18]. Then we retrieved lncRNA-protein interactions by manually filtering these interactions not involving lncRNAs. We also gave up the lncRNAs that interacts only one protein, because such interactions cannot be validated by leave-one-out cross validation (LOOCV). Finally, we got an lncRPI dataset that consists 4467 lncRPIs, involving 1050 unique lncRNAs and 84 unique proteins.

### Overview of our method

**Fig. 1:**
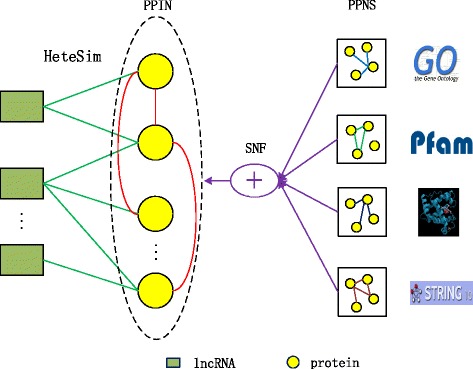
The pipeline of our method

The pipeline of our method is shown in Fig. 1. The rectangles represent lncRNAs and circles represent proteins. On the right side of figure, four squares mean different protein-protein similarity networks (PPSNs) with different similarity metrics. We use Similarity Network Fusion (SNF) algorithm to fuse them to get a more informative PPSN. Then we construct a heterogenous lncRNA-protein network based on known lncRPIs and the fused PPSN. The green solid lines are known lncRPIs and red solid lines are the similarity of proteins. Finally, we perform the HeteSim algorithm on the heterogenous network to predict novel lncRPIs.

As most lncRNAs do not show the same pattern of high interspecies conservation as protein-coding genes [19]. To avoid difficulties caused by low conservation, we predict lncRNA-protein interactions from the perspective of interaction network. However, with a few lncRNAs’ crosstalk reported, our lncRNA-protein interaction network considers of only a lncRNA-protein sub-network and a protein-protein sub-network. We adopt the HeteSim [14] to predict novel lncRPIs on the heterogenous network.

### Protein-protein similarity computation

Many sources are available to evaluate the similarity between proteins. In this paper, we calculate protein-protein similarity by using protein sequences, protein domains, protein functional annotations (or GO terms), and PPI confidence data from the STRING database v10.0 [20]. As protein sequences, domains and GO terms are different types of data with different biological implications, we employ different methods to compute the similarity between any pair of such data items. The computation results in four similarity matrices, denoted as Seqs, Pfam, Go and STRING, corresponding to four *protein-protein similarity networks* (PPSNs).

#### Sequence similarity (Seqs)

Protein sequences are obtained from the UniProt database [21]. We compute the sequence similarity between two proteins using a normalized version of Smith-Waterman score [22]. The normalized Smith-Waterman score between proteins *p*_*i*_ and *p*_*j*_ is $nsw(p_{i}, p_{j})=\frac {sw(p_{i}, p_{j})}{\sqrt {sw(p_{i}, p_{i})}\sqrt {sw(p_{j}, p_{j})}}$ where *s* *w*_(.,.)_ means the original Smith-Waterman score. By applying this operation to any protein pair of *p*_*i*_ and *p*_*j*_, we can obtain their sequence similarity as *S* *S*(*p*_*i*_,*p*_*j*_)=(*n* *s* *w*(*p*_*i*_,*p*_*j*_)+*n* *s* *w*(*p*_*j*_,*p*_*i*_))/2.

#### Functional annotation semantic similarity (Go)

GO annotations are downloaded from the GO database [23]. Semantic similarity between any pair of proteins is calculated based on the overlap of the GO terms associated with the two proteins [24]. All three types of GO are used in the computation as similar RNAs are expected to interact with proteins that act in similar biological processes, or have similar molecular functions or reside in similar cell compartments. We compute the Jaccard value [25] with respect to the GO terms of each pair of proteins as their similarity. The Jaccard score between two term sets *t*_*i*_ and *t*_*j*_ of proteins *p*_*i*_ and *p*_*j*_ is defined as $\frac {|t_{i} \cap t_{j}|}{ |t_{i} \cup t_{j}|}$, which is the ratio of the number of common terms between proteins *p*_*i*_ and *p*_*j*_ to the total number of terms of *p*_*i*_ and *p*_*j*_, which is used as the functional annotation semantic similarity *F* *S*(*p*_*i*_,*p*_*j*_) of proteins *p*_*i*_ and *p*_*j*_.

#### Protein domain similarity (Pfam)

Protein domains are extracted from the Pfam database [26]. Each protein is represented by a domain fingerprint (binary vector) whose elements encode the presence or absence of each retained Pfam domain by 1 or 0, respectively. We compute the Jaccard value of any two proteins *p*_*i*_ and *p*_*j*_ with their domain fingerprints as their similarity *D* *S*(*p*_*i*_,*p*_*j*_).

#### STRING similarity (String)

STRING is a database of known and predicted interactions which currently covers 9643763 proteins from 2031 organisms [20]. It provides a confidence score for the interaction of any two interacting proteins, and the highest score is 999. We use the confidence scores to evaluate the similarities between interacting proteins. Formally, for proteins *p*_*i*_ and *p*_*j*_, their similarity is *S* *t* *r* *i* *n* *g*(*p*_*i*_,*p*_*j*_) = *c* *o* *n* *f* *i* *d* *e* *n* *c* *e*_*s* *c* *o* *r* *e*(*p*_*i*_,*p*_*j*_)/999.

### Fusing protein-protein similarity networks

As each protein-protein similarity matrix (network) computed above may contain noise, here we fuse these four matrices (network) to get a more informative and reliable matrix (or network). The similarity network fusion (SNF) algorithm [16] is employed. SNF can derive useful information even from a small number of samples, and is robust to noise and data heterogeneity. It is a nonlinear message-passing based method that iteratively updates each network and makes it more and more similar to the other networks.

A PPSN can be represented as a graph *G*=(*V*,*E*) where *V*={ *v*_1_,*v*_2_,⋯,*v*_*n*_} corresponds to the set of proteins in the network and *E* corresponds to the set of edges, each of which has a similarity weight. We denote the corresponding similarity matrix as ***W*** where ***W***(*i*,*j*) is the similarity between proteins *v*_*i*_ and *v*_*j*_. To compute the fused matrix (network) from the four protein similarity matrices, we define a full and sparse kernel on each matrix. The full kernel is a normalized weight matrix ***P***=***D*** ^−1^ ***W***, ***D*** is a diagonal matrix and $\boldsymbol {D}(i, i)=\sum _{j}\boldsymbol {W}(i, j)$. To avoid numerical instability since ***P*** involves self-similarities on the diagonal entries of ***W***, a better normalization is as follows [16]:

1 $$ \boldsymbol{P}(i, j)=\left\{ \begin{aligned} \frac{\boldsymbol{W}(i, j)}{2\sum_{k\neq i}\boldsymbol{W}(i,k)}&,j\neq i \\ 1/2 &,j=i \end{aligned} \right.  $$

We denote protein *v*_*i*_’s neighbours as *N*_*i*_ and use *k* nearest neighbors (kNN) to measure the local affinity as follows:

2 $$ \boldsymbol{S}(i, j)=\left\{ \begin{aligned} \frac{\boldsymbol{W}(i, j)}{\sum_{k \in N_{i}}\boldsymbol{W}(i,k)}&,j\in N_{i} \\ 0 &, otherwise \end{aligned} \right.  $$

We think that the similarities between a protein and its neighbours are more reliable than the similarities between the protein with remote ones. Through graph diffusion, the similarities can be disseminated to remote proteins. Matrix ***P*** carries all information of the protein-protein similarity network and ***S*** carries local similarity information of the network. Then, we can do iterative computation as follows:

3 $$ \boldsymbol{P}_{t}^{(i)} = \boldsymbol{S}^{(i)} \times \left(\frac{\sum_{k\neq i}{\boldsymbol{P}_{t-1}^{(k)}}}{m-1}\right) \times (\boldsymbol{S}^{(i)})^{T}, i=1, 2, 3, 4,  $$

where $\boldsymbol {P}_{t}^{(i)}$ is the *i* ^*th*^ similarity matrix (network) after *t* (≥0) iterations, ***S*** ^(*i*)^ is the kNN matrix of the *i* ^*th*^ similarity matrix (network). *m* is the number of PPSNs used, here *m*=4. As ***S*** is the kNN neighbour matrix of ***P***, it contains the most important information of ***P*** and also alleviates the noise effect in ***P***. At each iteration, each similarity matrix (network) can get reliable information from the other similarity matrices (networks) and also updates itself with the other similarity matrices (networks). After *t* iterations, the fused matrix (network) is computed as follows:

4 $$ \boldsymbol{P} = \left(\sum\limits^{m}_{i=1} \boldsymbol{P}_{t}^{(i)}\right)/m.  $$

Note that we normalize matrix ***P***_*t*_ after each iteration to ensure the matrix is full rank and each protein is more similar to itself than the other proteins.

### Evaluating relevance score in a lncrna-protein network

With the known lncRPIs and the fused protein-protein similarity network, we build a lncRNA-protein heterogenous network, on which a random walk model HeteSim [14] is employed to infer new lncRPIs. HeteSim is to evaluate the relevance between a pair of lncRNA and protein, and a large relevance score means a high possibility that the lncRNA and protein interacts.

Given a schema *S*=(*A*,*R*) where *A* is a set of object types and *R* is a set of relationships. A lncRNA-protein network is defined as a directed graph ***G***=(***V***,***E***) with an object-type mapping function *ϕ*:***V***→*A* and a edge-relationship mapping function *ψ*:***E***→*R*. Each object *v*∈***V*** belongs to one particular object type *ϕ*(*v*)∈*A*, and each link *e*∈***E*** belongs to a particular relationship *ψ*(*e*)∈*R*. The schema *S* depicts the object types and the relationships existing among object types. For example, a relationship existing from type *A* to type *B*, denoted as $A \xrightarrow {R} B$, *A* and *B* are termed the source type and target type of relationship *R*. In this paper, there are two object types: lncRNA and protein, and three possible relationships: lncRNA-protein, protein-protein, and lncRNA-lncRNA. Here, we consider only the former two relationships. An object may be a concrete protein or lncRNA, and two objects can be connected via different paths that have different meanings.

In the heterogeneous network, a *relevance path* along a sequence of object types *A*_1_,*A*_2_,⋯,*A*_*l*+1_ can be denoted as $A_{1}\xrightarrow {R_{1}}A_{2}\xrightarrow {R_{2}}\cdots \xrightarrow {R_{l}}A_{l+1}$, the *composite relationship* between *A*_1_ and *A*_*l*+1_ is denoted as *R*=*R*_1_∘*R*_2_∘⋯∘*R*_*l*_ where ∘ denotes the relationship between two object types. For two objects *o*_1_ and *o*_2_ with a composite relationship *R*=*R*_1_∘*R*_2_∘⋯∘*R*_*l*_, HeteSim iteratively evaluates the relevance score between them as follows:

5 $${\kern-16.5pt} {{\begin{aligned} HeteSim(o_{1}, o_{2}|R_{1}\circ R_{2} \circ \cdots \circ R_{l}) =\frac{1}{|O(o_{1}|R_{1})||I(o_{2}|R_{l})|}\\ \sum_{i=1}^{|O(o_{1}|R_{1})|}\sum_{j=1}^{|I(o_{2}|R_{l})|} HeteSim(O_{i}(o_{1}|R_{1}), I_{j}(o_{2}|R_{l})|R_{2} \circ \cdots \circ R_{l-1}), \end{aligned}}}  $$

where *O*(*o*_1_|*R*_1_) is the out-neighbours of *o*_1_ based on relationship *R*_1_, *I*(*o*_2_|*R*_*l*_) is the in-neighbours of *o*_2_ based on relationship *R*_*l*_, *O*_*i*_(*o*_1_|*R*_1_) / *I*_*j*_(*o*_2_|*R*_*l*_) indicate the *i* ^*t*^ *h* / *j* ^*t*^ *h* object in *O*(*o*_1_|*R*_1_) / *I*(*o*_2_|*R*_*l*_), |·| means the size of a set.

As we consider only *lncRNA-protein relationship* (*lp* in short) and *protein-protein relationship* (*pp* in short), so we have

6 $${} {{HeteSim({lncRNA}_{i}, p_{j}|lp)=\left\{ \begin{array}{ll} 1 & \text{if they interact with each other;}\\ 0 & \text{otherwise}. \end{array} \right.}}  $$

7 $$ HeteSim(p_{i}, p_{j}|pp) = sim(p_{i}, p_{j}).  $$

For relationship $A \xrightarrow {R} B$, we define *U*_*AB*_ is a normalized adjacent matrix along the row vector between type *A* and type *B* based on relationship *R*. What is more, *V*_*AB*_ is the normalized matrix along the column vector, which is the transition probability matrix of $B \xrightarrow {} A$ based on inverse relationship *R* ^−1^. So, we can get $U_{AB} = V_{BA}^{'}\phantom {\dot {i}\!}$ and $V_{AB} = U_{BA}^{'}\phantom {\dot {i}\!}$ [14], where $V_{BA}^{'}$ is the transpose of *V*_*BA*_. Given a relevance path *P*=*A*_1_ *A*_2_⋯*A*_*l*+1_. The reachable probability matrix *PM* for path *P* is defined as $PM_{P}=U_{A_{1}A_{2}}U_{A_{2}A_{3}} \cdots U_{A_{l}A_{l+1}}\phantom {\dot {i}\!}$ (*PM* in short). *P* *M*(*i*,*j*) represents the probability of object *i*∈*A*_1_ reaching object *j*∈*A*_*l*+1_ based on path *P*. So the relevance between objects in *A*_1_ and *A*_*l*+1_ based on the relevance path *P* is:

8 $${} \begin{aligned} &HeteSim(A_{1}, A_{l+1}|P)\\ &\qquad= HeteSim(A_{1}, A_{l+1}|P_{L}P_{R})\\ &\qquad= U_{A_{1}A_{2}} \cdots U_{A_{mid-1}M}V_{MA_{mid+1}} \cdots V_{A_{l}A_{l+1}} \\ &\qquad= U_{A_{1}A_{2}} \cdots U_{A_{mid-1}M}U_{A_{mid+1}A_{M}}^{'} \cdots U_{A_{l+1}A_{l}}^{'} \\ &\qquad= U_{A_{1}A_{2}} \cdots U_{A_{mid-1}M}(U_{A_{l+1}A_{l}}\cdots U_{A_{mid+1}A_{M}})^{'} \\ &\qquad= PM_{P_{L}}PM_{P_{R}^{-1}}^{'} \end{aligned}  $$

where *M* is the middle position node type of *A*_1_ and *A*_*l*+1_. So above equation shows the inner product of matrices of two probability distributions that *A*_1_ reaches *M* and *A*_*l*+1_ reaches *M*.

For two instances *o*_1_ and *o*_2_ of type *A*_1_ and type *A*_*l*+1_, we can get their normalized relevance score:

9 $$ HeteSim(o_{1}, o_{2}|P) = \frac{PM_{P_{L}}\left(o_{1}, :\right)PM_{P_{R}^{-1}}^{'}\left(o_{2},:\right)}{\sqrt{\|PM_{P_{L}}(o_{1}, :)\|\|PM_{P_{R}^{-1}}^{'}(o_{2},:)\|}}  $$

where $PM_{P_{L}}(o_{1}, :)$ is the row that object *o*_1_ lies in the matrix $PM_{P_{L}}$ [14].

## Results and discussion

In our experiments, the leave-one-out cross validation (LOOCV) is used to evaluate the proposed method. The baseline is the method proposed by Yang et al. [13] where PPIs were modeled as a binary network. As for our method, there are 15 settings: 4 settings of using only one similarity matrix, Seqs, Pfam, Go and STRING, respectively; 6 settings of fusing two similarity matrices, corresponding to Seqs+Pfam, Seqs+Go, Seqs+String, Pfam+Go, Pfam+String, and Go+String; 4 settings of fusing three similarity matrices, including Seqs+Pfam+Go, Seqs+Pfam+String, Seqs+Go+String, and Pfam+Go+String; and 1 setting of fusing the four similarity matrices, i.e., Seqs+Pfam+Go+String. For reference simplicity, we denote the baseline method as *Binary*, and denote our method under different settings by the setting names, such Seqs, Seqs+Pfam, Seqs+Pfam+Go, Seqs+Pfam+Go+String, etc. Actually, the String case of our method is roughly similar to the LPIHM method [15]. So essentially we compare our method with both the method in [13] and the LPHIM method in [15]. Because HeteSim is a path-constrained relevance measure, the selection of path is very important. In Yang et al.’s work, they chose *lncRNA-protein-protein* (LPP) as their relevance path and achieved better performance than other pathes. In our work, we also choose it as the relevance path.

To evaluate the prediction performance, the *receiver operating characteristic* (ROC) curve is generated for each experimental setting, and AUC (the area under the ROC curve) is calculated, which is widely used in assessing prediction performance and its value falls between 0 and 1. The maximum value 1 means a perfect prediction, and 0.5 means a random guess.

**Fig. 2:**
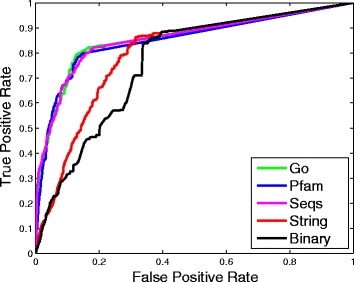
ROC curves of Binary and our method using only one of different similarity matrices

We first compare our method with only one similarity matrix to the baseline method *Binary*, the results are presented in Fig.2. The black solid line is the ROC curve of *Binary* and the other colored lines are the ROC curves of our method with different similarity matrices. In Fig.2, we can see that the performance of *String* is better than that of *Binary*, which shows that weighted PPI network is more helpful than binary PPI network. Moreover, the results of *Go*, *Pfam* and *Seqs* are all better than that of *String*. This may be because String is less reliable than the other similarity networks.

**Fig. 3:**
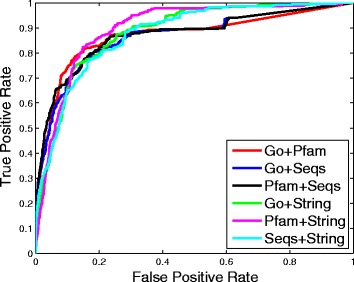
ROC curves of our method when fusing two different matrices

**Fig. 4:**
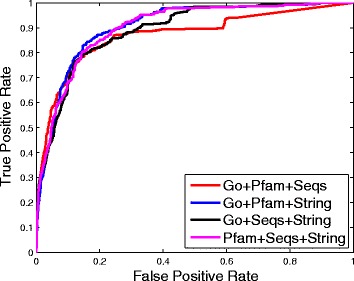
ROC curves of our method when fusing three different matrices

**Fig. 5:**
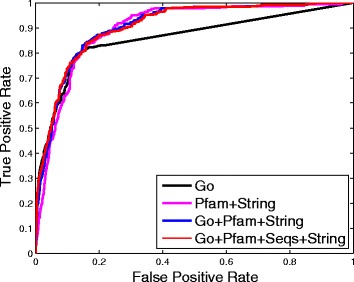
ROC curves of the best performance when using one matrix and fusing two, three and four matrices, respectively

We then compare the performance of our method under different experimental settings, the results are shown in Figs. 3, 4 and 5.

First, we consider the cases of fusing two different similarity matrices, their corresponding ROC curves are presented in Fig. 3. Each color curve indicates the ROC curve of our method of fusing two specific similarity matrices. We can see that fusing the String similarity matrix with any other similarity matrix can achieve better performance than fusing any other two similarity matrices. This may be because STRING database has many PPIs that contain much complementary information to the other similarity matrices. We achieve the best performance when fusing the Pfam similarity matrix and the String similarity matrix.

Then, we check the cases that fuse three different similarity matrices, their ROC curves are shown in Fig. 4. We get the best performance when fusing Go, Pfam and String similarity matrices. Similar to Fig. 3, we can see that the performance when fusing the String similarity matrix with any two other similarity matrices is better than that of Go+Pfam+Seqs.

Finally, we consider the case that fuses all four similarity matrices, and present its ROC curve in Fig. 5. For the convenience of comparison, in Fig. 5 we also plot the best results when using one similarity matrix, and fusing two and three different similarity matrices. It can be seen that the performance of Go+Pfam+Seqs+String is better than the performance of the other settings (Go, Pfam+String, Go+Pfam+String). This illustrates that network fusion can really extract complementary information from different networks to achieve better prediction performance.

**Fig. 6:**
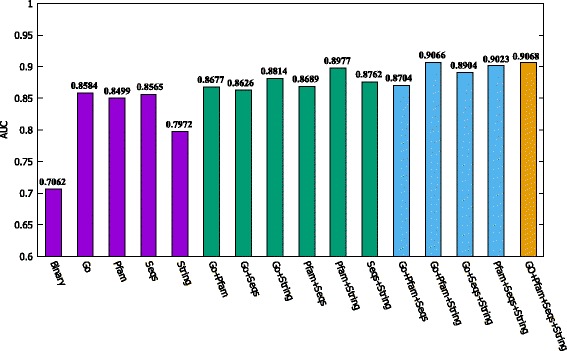
The AUC values of the baseline method and our method under different settings

To more clearly compare the baseline method *Binary* and our method under different settings, we give all of their AUC values in Fig. 6. Here, the bars of similar color means using the same number of similarity matrices. We can see that 1) all the AUC values of our method under different settings are larger than that of using a binary PPI network; 2) As more matrices are fused, the AUC value becomes larger. For example, the AUC value of Go+Pfam+String is 0.9066, which is bigger than the AUC values of Go+Pfam, Go+String and Pfam+String. And when fusing all the four matrices, the corresponding AUC value is the largest (0.9068). This shows that by fusing multiple matrices we can get a more reliable and informative matrix or network.

## Conclusion

In this paper, we proposed a new approach to predicting lncRPIs by fusing four protein-protein similarity networks, which were computed with protein sequences, protein domains, protein functional annotations of GO, and the PPI confidence scores from the STRING database. The similarity network fusion (SNF) algorithm and the random walk on heterogeneous network model HeteSim were employed. Our experimental results show that the proposed method outperforms the existing method and those cases when using only one protein-protein similarity network. For future work, on the one hand, we will explore other advanced network fusion methods to fuse more available data sources for further boosting the performance of lncRPI prediction; On the other hand, we will include the lncRNA-lncRNA interactions into the prediction procedure.
